# A prospective cohort study of MTHFR C677T gene polymorphism and its influence on the therapeutic effect of homocysteine in stroke patients with hyperhomocysteinemia

**DOI:** 10.1186/s12883-020-01701-8

**Published:** 2020-04-11

**Authors:** Xiaoxia Du, Lin Xiao, Rong Sun, Kunpeng Li, Lin Liang, Luping Song, Zhizhong Liu

**Affiliations:** 1 Department of Neurorehabilitation, School of Rehabilitation Medicine, Capital Medical University, Beijing Bo’Ai Hospital, China Rehabilitation Research Center, Beijing, 100068 China; 2 Case Statistics Office, School of Rehabilitation Medicine, Capital Medical University, Beijing Bo’Ai Hospital, China Rehabilitation Research Center, Beijing, 100068 China; 3 Department of Clinical Laboratory, School of Rehabilitation Medicine, Capital Medical University, Beijing Bo’Ai Hospital, China Rehabilitation Research Center, Beijing, 100068 China

**Keywords:** Homocysteine, Stroke, Intracerebral hemorrhage, Methylenetetrahydrofolate reductase, Single nucleotide polymorphism

## Abstract

**Background:**

Hyperhomocysteinemia (HHCY) is a risk factor for cardiovascular and cerebrovascular diseases. The C677T 5, 10-methylenetetrahydrofolate reductase (MTHFR) gene polymorphism increases homocysteine (HCY) levels. This study analyzed the relationship between C677T MTHFR polymorphism and the therapeutic effect of lowering HCY in stroke patients with HHCY.

**Methods:**

Baseline data were collected from stroke patients with HHCY for this prospective cohort study. The C677T MTHFR genotype was detected by polymerase chain reaction-restriction fragment length polymorphism and the therapeutic effect to reduce HCY was compared.

**Results:**

Of 200 stroke patients 162 (81.0%) completed follow-up and were evaluated. Most of them responded well to treatment (103 cases, 63.5%), but 59 (36.4%) patients were in the poor efficacy group. There was a significant difference in terms of age (*P* < 0.001), hypertension (*P* = 0.041), hyperuricemia (*P* = 0.042), HCY after treatment (*P* < 0.001), and MTHFR genotype (*P* < 0.001) between the poor efficacy and effective groups, with increased frequency of the TT genotype in the poor efficacy group. Logistic regression showed that the T allele was associated with poor efficacy (OR = 0.733, 95%CI: 0.693, 0.862, *P* < 0.001). In the codominant model the TT genotype was associated with poor outcome (OR = 0.862, 95%CI: 0.767, 0.970, *P* = 0.017) and this was also the case in the recessive model (OR = 0.585, 95%CI: 0.462, 0.741, *P* < 0.001) but there was no association between CT and TT in the dominant model.

**Conclusions:**

The T allele and TT genotype of the MTHFR C677T polymorphism was associated with poor HCY reduction treatment efficacy in stroke patients with HHCY.

**Trial registration:**

The registration number of the clinical trial is ChiCTR1800020048. Registration date: December 12, 2018.

## Background

The global burden of stroke is extremely high, 16.9 million people suffer a stroke each year, and the aging and increasing number of the world’s population means stroke is increasing [1]. The traditional risk factors of stroke, such as hypertension, diabetes mellitus, hyperlipidemia, and atrial fibrillation have been studied extensively, and their underlying mechanisms for stroke are evident [2]. This has led to primary prevention measures that have been implemented to decrease the incidence of stroke in high income countries [1]. As stroke can lead to long-term disability, an early detection of risk factors and active control to effectively preventing the occurrence of stroke is particularly important [3]. Effective prevention is an important strategy to reduce the overall burden of stroke worldwide.

However, many other risk factors are less well understood. Homocysteine (HCY) is a less well studied non-traditional risk factor for stroke [4, 5]. Framingham’s offspring cohort study found that the predictive power of the score will be improved if four biomarkers, including homocysteine, were included in the Framingham Stroke Risk Score [6, 7]. Our previous studies found that elevated HCY was a risk factor for stroke in young people [8, 9]. The correlation between high HCY and stroke is stronger in younger individuals [9]. Patients with an abnormally high level of HCY, or hyperhomocysteinemia (HHCY), are considered to be at risk of cardiovascular and cerebrovascular diseases [10–12], but its mechanism is rather complex, and not fully understood. 60.6% of stroke patients have HHCY, which is associated with a low level of serum B12 [13]. There is indeed a significant positive correlation between HCY levels and ischemic stroke [14–17]. Intracerebral hemorrhage (ICH), which accounts for 10–15% of cases, has the highest mortality and morbidity rate among all strokes. HCY level may be an aggravating factor in atherosclerosis, which indirectly contributes to a high risk of ICH [18, 19]. High HCY concentrations may impair endothelial function, increase oxidative stress, impair methylation reactions, and alter protein structure [20]. Elevated HCY is affected by diet, nutrition, heredity, disease and drug factors [21, 22]. The contribution of genetic factors to the pathogenesis of stroke is demonstrated by the association between specific gene variants and stroke risk. However, due to conflicting results from different studies [13, 23, 24], the effects of these polymorphisms on the risk of stroke development are remain uncertain. It has been suggested that differences between studies are related to the heterogeneity of cerebral infarction [25–27].

Among genetic factors, the C677T gene polymorphism site of methylenetetrahydrofolate reductase (MTHFR) gene has become an important research locus [28]. MTHFR encodes methylenetetrahydrofolate reductase which is a rate-limiting enzyme for folic acid metabolism. Dietary folate converts to its active cofactor in HCY catabolism and catalyze the conversion of 5, 10-methylenetetrahydrofolate to 5-methyltetrahydrofolate. Thus, it plays an important role in folic acid metabolism, DNA methylation and repair [29]. Some studies have shown that individuals with C677T MTHFR genotype have doubled HCY levels in plasma compared with normal individuals [29]. The C677T mutation apparently modifies the association between HCY and stroke [30]. However it is not clear whether the C677T MTHFR polymorphism influences efforts to decrease HCY in patients with stroke and HHCY.

This study aimed to compare the relationship among C677T MTHFR gene polymorphism and the risk of stroke, and the therapeutic effect of lowering HCY in stroke patients with HHCY. The results should help reduce the family burden and economic burden of the stroke through standardized prevention and treatment strategies in stroke patients with HHCY.

## Methods

This prospective cohort study (ChiCTR1800020048) was approved by the institutional review board of China Rehabilitation Research Center (CRRC) (2016–018-1). Written informed consent was obtained from all the patients. The study was conducted in accordance with the principles of the Declaration of Helsinki.

### Patients

From March 2016 to December 2018, we recruited stroke patients with HHCY in the outpatients clinic and wards of the Department of Neurorehabilitation of CRRC. The inclusion criteria were as follows: 1) age ≥ 18 years; 2) patients with ischemic stroke (consistent with diagnostic criteria in the 2010 guidelines for the diagnosis and treatment of acute ischemic stroke in China [31]); patients with cerebral hemorrhage (diagnostic criteria at the Fourth National Conference on Cerebrovascular Diseases [32]); 3) patients with HHCY (HCY ≥ 15 μmol/L). The exclusion criteria were as follows: 1) patients with subarachnoid hemorrhage; patients with traumatic intracerebral hemorrhage; 2) pregnant or lactating women; 3) patients participated in or were participating in other clinical studies within 3 months; 4) patients with malignant tumors. The exit and termination criteria were: patients who could not complete the examination items as required or could not be followed up.

The clinical data of the patients were collected, including gender, age, history of hypertension, history of diabetes, history of lipid metabolism disorder, history of heart disease, and type of stroke.

### Detection of HCY and C677T polymorphism of the MTHFR gene

All the subjects were tested for HCY and MTHFR C677T gene polymorphism in the Laboratory Department of CRRC.

The detection and reference values of plasma HCY were as follows: all patients were tested for baseline HCY in the morning, 5 ml of fasting elbow venous blood was collected (heparin anticoagulation), and centrifuged for 10 min at 3000 r/min, and the plasma HCY concentration was measured by a chemiluminescence with a BIO-RAD automatic biochemical analyzer (S1000 PCR instrument) using the mindray reagent. The normal reference range set by the kit manual was 5-15 μmol/L [33], and if HCY is more than 15 μmol/L, HHCY should be considered. After 1 month of treatment, the patients were tested again.

The MTHFR C677T gene polymorphism was tested with the Baio MTHFR (C677T) gene detection kit using a BS-800 M S1000TM Thermal cycler. The C677T locus genotyping of MTHFR gene was performed by polymerase chain reaction-restriction fragment length polymorphism.

### Treatment

In this study, patients were given folic acid tablets 0.4 mg/day, vitamin B6 100mg with 3 times/day, methylcobalamin 500 μg with 3 times/day to reduce HCY. The data of Baseline levels and HCY levels after 1 month of treatment were collected. Patients with poor efficacy were those who failed to reach the normal range or whose reduction was less than 20%. The rest of the patients were considered as control groups. Patients with other diseases (hypertension, diabetes, etc.) were treated according to their original treatment plan.

### Statistical analysis

SPSS 21.0 (IBM Corp., Armonk, NY, USA) statistical software was used to analyze the research data. The measurement data were expressed as ^−^x ± s. T-test was used for comparison between the two groups, and analysis of variance (ANOVA) was used for multi-group comparison. Least significant difference (LSD) test was used for pairwise comparison, and the paired t-test was used for paired data. The count data were expressed as examples (percentages) and was compared by chi-square test. Regarding effective treatment as the outcome, logistic regression was used to analyze the correlation between different genotypes and outcomes (indicators with *P* < 0.05 of univariate analysis were selected for multivariate analysis). *P* < 0.05 was accepted as a significant difference.

## Results

### Baseline characteristics and HCY change after treatment

A total of 200 stroke patients were enrolled in the study. Of these, 11 cases (5.5%) withdrew from the study due to renal insufficiency, and 27 cases (13.5%) withdrew from the study due to hepatic insufficiency without a full course of regular treatment. A total of 162 patients (81.0%) completed the follow-up. Most of them responded well to folic acid tablets, vitamin B6 and methlcobalamin treatment, with a reduction of more than 20% or a reduction to the normal range and so they were grouped into the effective efficacy group (103 cases, 63.5%). The remaining 59 (36.4%) patients were grouped in the poor efficacy group (Table 1). There were significant differences between the groups in age (*P* < 0.001), hypertension (*P* = 0.041), hyperuricemia (*P* = 0.042), HCY after treatment (*P* < 0.001), and MTHFR genotype (*P* < 0.001).

**Table 1:** Baseline characteristics and homocysteine levels before and after treatment in stroke patients grouped according to treatment efficacy

	Poor efficacy group (*n* = 59)	Effective efficacy group (*n* = 103)	*P* value
Gender, male (%)	51 (86.4%)	87 (84.5%)	0.734
Age, years	46.51 ± 13.7	57.7 ± 13.3	< 0.001
Stroke types			
Hemorrhagic, n (%)	34 (57.6%)	45 (43.7%)	0.088
Ischemic, n (%)	25 (42.4%)	58 (56.3%)	
Hypertension, n (%)	55 (93.2%)	84 (81.6%)	0.041
Diabetes mellitus, n (%)	17 (28.8%)	38 (36.9%)	0.269
Abnormal lipid metabolism, n (%)	34 (57.6%)	75 (72.8%)	0.070
Hyperuricemia, n (%)	10 (16.9%)	7 (6.8%)	0.042
HCY, μmol/L			
Before treatment	26.49 ± 15.75	21.88 ± 18.99	0.116
After treatment	21.64 ± 10.81	12.42 ± 5.99	< 0.001
MTHFR C677T genotype			
CC, n (%)	1 (1.7%)	9 (8.7%)	< 0.001
CT, n (%)	10 (16.9%)	45 (43.7%)	
TT, n (%)	48 (81.4%)	49 (47.6%)	

### MTHFR polymorphism and HCY changes

The *P* value of the Hardy-Weinberg equilibrium test was 0.56, which indicated the genetic balance of the population. In the poor efficacy group of 59 cases, there were 48 cases of TT, 10 cases of CT; 1 case of CC. Within this group there were 12 patients with unstable HCY levels which are increased instead of being decreased, in which 11 cases were TT, and 1 case was CT (Table 2). HCY levels before and after treatments for each genotype are shown in Table 3. These results showed that there were significant differences in HCY levels among the genotype groups before (*P* = 0.049) and after (*P* = 0.002) treatment.

**Table 2:** Distribution of the three MTHFR C677T genotypes in patients with poor efficacy

Patients with poor efficacy	CC (*n* = 1)	TT (*n* = 49)	CT (*n* = 9)
Decrease< 20% (*n* = 47)	1	38	8
Unstable increase (*n* = 12)	0	11	1

**Table 3:** Homocysteine levels before and after treatment according to MTHFR C677T genotype

	CC (*n* = 10)	CT (*n* = 55)	TT (*n* = 97)	*P* value^*^
HCY before treatment, μmol/L	20.26 ± 6.60	18.90 ± 6.37	21.75 ± 8.19	0.049
HCY after treatment, μmol/L	14.02 ± 3.53	12.98 ± 5.01 ^ab^	19.45 ± 11.32 ^ab^	0.002
*P* value^**^	0.535	0.328	0.211	

^*^ Analysis of variance was used for comparison among genotypes

^**^ Paired sample t-test was used for comparison between baseline and 1 month after treatment

^ab^ Different letters indicate statistical significance. Least significant difference (LSD) test was used for pairwise comparison. CCvsCT *p* = 0.816; CC vs TT *p* = 0.215; CT vs TT *p* = 0.001

Logistic regression analysis was carried out with adjustment for age, history of hypertension and hyperuricemia for the outcome of poor efficacy, and the results showed that the T allele was associated with poor efficacy (OR = 0.733, 95%CI: 0.693, 0.862, *P* < 0.001). In the codominant model the TT genotype was associated with poor outcome (OR = 0.862, 95%CI: 0.767, 0.970, *P* = 0.017) and this was also the case in the recessive model (OR = 0.585, 95%CI: 0.462, 0.741, *P* < 0.001) but there was no association of between CT and TT in the dominant model (Table 4).

**Table 4:** Logistic regression analysis of MTHFR C677T in poor efficacy cases and controls

	Cases, n (%)	Controls, n (%)	OR (95% CI)	*P* value
**Allele**				
C	63 (30.6)	12 (10.2)	1.000 (referrence)	–
T	143 (69.4)	106 (89.8)	0.773 (0.693, 0.862)	< 0.001
**Model type**				
Codominant				
CC	9 (8.7)	1 (1.7)	1.000 (referrence)	–
CT	45 (43.7)	10 (16.9)	0.917 (0.734, 1.144)	0.460
TT	49 (47.6)	48 (81.4)	0.862 (0.767, 0.970)	0.017
Dominant				
CC	9 (8.7)	1 (1.7)	1.000 (referrence)	–
CT + TT	94 (91.3)	58 (98.3)	0.928 (0.867, 0.994)	0.095
Recessive				
CC + CT	54 (52.4)	11 (18.6)	1.000 (referrence)	–
TT	49 (47.6)	48 (81.4)	0.585 (0.462, 0.741)	< 0.001

## Discussion

MTHFR involved in folate metabolism has two variants, C677T and A1298C [34]. Racial-ethnic differences in the distribution of the C677T polymorphism are well-described [35, 36]. Carrier rates of the 677 T polymorphism differ across white, Asian, and black ethnic groups. This study aimed to analyze the relationship between C677T MTHFR polymorphism and the therapeutic effect of lowering HCY in stroke patients with HHCY. Results from previous studies have shown that the TT genotype of the MTHFR gene in Asian countries had a greater impact on HCY levels [15, 37, 38]. The TT genotype of C677T polymorphism in the MTHFR gene contributes to the genetic susceptibility of acute ischemic stroke inpopulation of Singapore [39]. The frequency of TT genotype of MTHFR gene in this group of stroke patients was up to 57.92% (117). TT genotype is more frequently seen in Asian compared with that of white individuals, and least common in those of African ethnic origin [29], However, ethnic subgroups may in turn exhibit different carrier rates or environmental susceptibility to disease-specific morphisms, which may in part explain ethic and regional differences in stroke risk [35]. These results suggest that race and genetic factors influence the incidence of disease. The results of this study showed that overall 59.9% of the stroke patients had the TT genotype. There were no significant differences between ischemic stroke group and hemorrhagic stroke group in the comparison of TT genotype (60.9% vs57.5%), but the percentage of patients with the TT genotype was much higher in the poor treatment efficacy group than that of patients who achieved a good reduction in HCY levels (81.4 47.6%). This shows the importance of early detection of HCY in the Chinese population with the TT genotype of MTHFR gene.

vitamin B, especially methylcobalamin, can effectively reduce plasma HCY. The most commonly used regimen is the combined supplementation of folic acid (vitamin B9), vitamin B12 and vitamin B6 [40]. Folic acid needs to be combined with vitamin B12 and vitamin B6 to reduce the risk of stroke effectively [41–43]. Previous studies suggested that 0.8 mg/d of folic acid have the best effect on reducing HCY [31]. The Framingham investigators and others have demonstrated reductions in total HCY following population folic acid grain fortification programs [44]. In areas where grain is not fortified, folic acid can slightly reduce stroke risk, However, there was no evidence that long-term use of larger doses of folic acid could further improve efficacy, and its safety is worthy of attention. In clinical practice, folic acid tablets, vitamin B6 and methylcobalamin are used to treat HHCY. According to the US guidelines, the supplementation of three kinds of compound vitamin B could reduce HCY and reduce the risk of stroke in HHCY [41, 45]. A daily multivitamin preparations with adequate vitamin B6 and vitamin B12, and folate (400μg/d) are reasonable to reduce the level of homocysteine [46]. The Chinese guidelines for Secondary Prevention of Ischemic Stroke and Transient Ischemic Attack (TIA) recommend supplementation of folic acid, vitamin B6 and vitamin B12 to be used to reduce HCY in patients with recent ischemic stroke or TIA and mild to moderate homocysteine elevation [31]. Most of the patients in this study responded well to folic acid tablets, vitamin B6 and methlcobalamin therapy. Patients affected more by genetic factors were less responsive to folic acid tablets, vitamin B6 and methlcobalamin and needed longer medication cycles. Literatures have shown that HHCY patients with TT genotype could still benefit from folic acid supplementation despite normal folic acid and vitamin B12 levels [47], but there are few studies on how to adjust treatment in a few patients with elevated levels. Elevated HCY is influenced by many factors such as diet, heredity, and drugs [48]. Further study is required to analyze genetic subgroups within large clinical trials of HHCY therapy. Young individuals with elevated HCY and people with high blood pressure may particularly benefit from lowering HCY [49].

Several limitations of this study must be acknowledged. This study was a single-center study with a relatively small sample size and only one-month follow-up time. For HHCY patients with TT genotype, methods to controlling the abnormal increases of HCY(the dosage, method, type and cycle of the drugs)need to be studied,and these are our future research direction. Multi-center clinical trials using larger sample sizes and better design will be needed to validate our results.

## Conclusions

In conclusion, the TT genotype of MTHFR C677T was common in this Chinese stroke population and was an independent risk factor for poor efficacy of HCY lowering treatment.

## Data Availability

The datasets used and/or analyzed during the current study are available from the corresponding author on reasonable request.

## Abbreviations

HHCY: Hyperhomocysteinemia
HCY: Homocysteine
ICH: Intracerebral hemorrhage
CRRC: China Rehabilitation Research Center
MTHFR: The 5, 10-methylenetetrahydrofolate reductase
OR: Odds ratio
CI: Confidence interval
LSD: Least significant difference
